# Vaccine confidence mediates the association between a pro-social pay-it-forward intervention and improved influenza vaccine uptake in China: A mediation analysis

**DOI:** 10.1016/j.vaccine.2023.11.046

**Published:** 2024-01-12

**Authors:** Wenwen Jiang, Chunlei Lu, Xumeng Yan, Joseph D. Tucker, Leesa Lin, Jing Li, Heidi J. Larson, Wenfeng Gong, Dan Wu

**Affiliations:** a West China School of Public Health and West China Fourth Hospital, Sichuan University, Chengdu, China; b School of Public Health of Nanjing Medical University, Nanjing, Jiangsu, China; c University of North Carolina Project–China, Guangzhou, China; d SESH (Social Entrepreneurship to Spur Health) Team, Guangzhou, China; e School of Medicine, University of North Carolina at Chapel Hill, Chapel Hill, NC, USA; f Faculty of Infectious and Tropical Diseases, London School of Hygiene & Tropical Medicine, Room 360, Keppel St, London WC1E 7HT, UK; g Department of Infectious Disease Epidemiology, London School of Hygiene & Tropical Medicine, London, UK; h Laboratory of Data Discovery for Health Limited (D24H), Hong Kong Science Park, Hong Kong Special Administrative Region; i WHO Collaborating Centre for Infectious Disease Epidemiology and Control, School of Public Health, LKS Faculty of Medicine, The University of Hong Kong, Hong Kong Special Administrative Region; j Institute for Health Metrics and Evaluation, University of Washington, Seattle, USA; k China Country Office of the Bill and Melinda Gates Foundation, China; l Department of Social Medicine and Health Education, School of Public Health of Nanjing Medical University, Nanjing, Jiangsu, China

**Keywords:** Pay-it-forward, Vaccine uptake, Vaccine confidence, Mediation analysis

## Abstract

**Introduction:**

A Chinese clinical trial has demonstrated that a prosocial pay-it-forward intervention that offered subsidized vaccination and postcard messages effectively increased influenza vaccine uptake and vaccine confidence. This secondary analysis explored the potential mediating role of vaccine confidence on the association between a pay-it-forward intervention and influenza vaccine uptake, and how this might vary by individual annual income levels.

**Methods:**

Data from 300 participants (150 standard-of-care and 150 pay-it-forward participants) were included in the analysis. We conducted descriptive analysis of demographic and vaccine confidence variables. Multivariable regression and mediation analysis on interventions, vaccine confidence and vaccine uptake were conducted. A sub-group analysis was conducted to further understand whether associations between these variables vary by income levels (<=$1860 or >$1860).

**Results:**

The pay-it-forward intervention was significantly associated with greater levels of perceived influenza vaccine importance (adjusted odds ratio (aOR) = 3.60, 95 %CI: 1.77–7.32), effectiveness (aOR = 3.37, 95 %CI: 1.75–6.52) and safety (aOR = 2.20, 95 %CI: 1.17–4.15). Greater perceived influenza vaccine importance was associated with increased vaccine uptake (aOR = 8.51, 95 %CI: 3.04–23.86). The indirect effect of the pay-it-forward intervention on vaccination was significant through improved perceived influenza vaccine importance (indirect effect_1_ = 0.07, 95 %CI: 0.02–0.11). This study further revealed that, irrespective of the individual income level, the pay-it-forward intervention was associated with increased vaccine uptake when compared to the standard-of-care approach.

**Conclusions:**

Pay-it-forward intervention may be a promising strategy to improve influenza vaccine uptake. Perceived confidence in vaccine importance appears to be a potential mediator of the association between pay-it-forward and vaccine uptake.

## 1 Introduction

Seasonal influenza is a major global public health concern [1]. The World Health Organization (WHO) estimates that influenza causes approximately 3–5 million severe cases and 290,000–650,000 deaths annually [2], [3]. Vaccination is the most cost-effective preventive measure. However, China has a low influenza vaccination rate among priority populations, including children at 11.9 % and older adults at 21.7 % [4]. Lack of vaccine confidence and public funding may be important contributing factors [5]. Vaccine confidence is encompassed in measures related to perceived vaccine safety, effectiveness, and importance [6], [7], [8], [9], [10]. Previous evidence suggested that individuals who had higher perceived confidence are more likely to receive a vaccine [11], [12], [13], [14], [15].

Vaccine confidence is driven by a mix of psychological, sociocultural factors [16], including community engagement [17], trust in health providers [18], and individual socioeconomic status [16]. Dissemination of false information on the Internet further undermines public confidence [19]. However, few studies have focused on improving vaccine confidence. One study indicated that education increased COVID-19 vaccine uptake and confidence in Canadian [20]. Currently, some ongoing studies are exploring innovative interventions to improve vaccine confidence [21], [22]. But none of these studies have examined the association between interventions, vaccine confidence and vaccine uptake.

Our research team implemented a pay-it-forward intervention to improve influenza vaccination in which one individual received a free influenza vaccine as a community gift and was offered an opportunity to donate to support another person to receive the same service. Pay-it-forward involving public engagement and community kindness, was proven to have increased influenza vaccine uptake compared to a self-paid vaccination approach [23]. However, our previous analysis primarily focused on the effects of the intervention on vaccine uptake and vaccine confidence as separate outcomes and did not attempt to examine the potential associations between these variables. Additionally, while pay-it-forward involved financial support, whether the associations between these variables vary by level of annual income remains unclear. This secondary analysis aimed to examine 1) the potential mediating roles of vaccine confidence on the association between pay-it-forward intervention and influenza vaccine uptake via a mediation analysis; and 2) potential varying associations between the pay-it-forward intervention and vaccine uptake by different individual annual income levels via a sub-group analysis. We hypothesized that the pay-it-forward intervention might be associated with vaccine uptake behaviors through potential mediators of vaccine confidence; and associations between the intervention and vaccine uptake/vaccine confidence may vary by level of individual annual income(≤$1860 or >$1860).

## 2 Methods

### 2.1 Study design

We conducted a secondary analysis using data from a parent quasi-experimental trial study that assessed the effectiveness of a pay-it-forward intervention against a standard-of-care self-paid vaccination arm [23]. Data were collected between September 21, 2020 and March 3, 2021 in Guangdong Province. The parent study comprised two intervention arms - a standard-of-care arm and a pay-it-forward arm. The parent study adopted a quasi-experimental design for pragmatic reasons. Due to the overwhelming workload during COVID-19 pandemic, community healthcare workers had limited willingness and capacity to help implement a randomized trial. Instead, recruited participants were chronologically allocated into the two study arms. Participants in the standard-of-care arm had to pay the standard market price of $8·5-23·2 for their vaccines, whereas participants in the pay-it-forward arm received a free influenza vaccination and a postcard message from a local group and were then asked if they would like to voluntarily donate any amount of money or write postcards to support vaccination for subsequent individuals. All participants received an introductory pamphlet about influenza and vaccination. A total of 300 individuals, 150 children (aged between 6 months and 8 years, via caregivers) and 150 older people (≥60 years old) were recruited in our study, with 75 children and 75 older people in each arm. Caregivers of children and older participants completed a questionnaire survey and then determined whether their children or older adults themselves wanted to receive a vaccination after the intervention in each arm.

### 2.2 Variable selection

#### 2.2.1 Sociodemographic characteristics

The final questionnaire survey administered in our study collected information on sociodemographic characteristics of participants (i.e., caregivers or older people) including study site (Yangshan, Zecheng, or Tianhe), sex of participant (male/female), age, educational level (primary school, middle school, and undergraduate or college), occupation (unemployed, peasant or employed), annual income (≤$1860 or >$1860), marital status (live alone/engaged or married), and participants’ attitudes towards vaccine confidence (importance, effectiveness and safety). Moreover, we considered the question “Is price of the vaccine a barrier for your child and/or older individuals in your family to get the influenza vaccine? (Yes/No)” as a proxy measure of sensitivity to vaccine costs (Yes/No). Participants who answered “Yes” were considered cost-sensitive, while those who answered “No” were considered cost-insensitive.

#### 2.2.2 Independent variable

The study participants were assigned to either the standard-of-care arm or the pay-it-forward arm based on their enrollment order. Intervention arm was treated as the independent variable in this secondary analysis.

#### 2.2.3 Mediators

Vaccine confidence in safety, importance, and effectiveness were hypothesized as potential mediators in this analysis. We measured vaccine confidence by adapting existing Vaccine Confidence Index ^TM^ scales for influenza vaccination in China [6], [24]. Vaccine confidence was assessed by the degree to which respondents agreed with the following statements on a five-point Likert scale: “In general, I think the influenza vaccine is important,” “In general, I think the influenza vaccine is safe,” “In general, I think the influenza vaccine is effective.” Based on prior vaccine confidence categorization in the literature [6], [25], the responses to the three statements were recoded into binary variables - agree (including “strongly agree” and “agree”) and disagree (including “strongly disagree”, “disagree”, and “unsure”). “Unsure” was categorized as disagree because the expression of uncertainty is generally interpreted as a form of disagreement in Chinese culture [26].

#### 2.2.4 Dependent variable

Vaccine uptake (Yes/No) among children and older adults was treated as the dependent variable. Data for the dependent variable was obtained from clinical vaccination records.

### 2.3 Statistical analysis

First, descriptive analysis was used to describe sample characteristics, including sociodemographic characteristics (study site, age group, sex, age, educational level, occupation, annual income, marital status, cost-sensitivity) and vaccine confidence (safety, importance and effectiveness).

Second, we employed multivariable logistic regression model to examine the association between pay-it-forward and vaccine uptake, controlling for sociodemographic variables (study site, age group, sex, age, educational level, occupation, annual income, marital status, cost-sensitivity). We also used multivariable logistic regression models to test the associations between pay-it-forward and vaccine confidence including safety, importance and effectiveness.

Third, we conducted sub-group analysis based on level of individual annual income to determine if the association between pay-it-forward and vaccine uptake is still present within sub-groups with different income-levels. We categorized the participants into two sub-groups based on the cut-off value for defining low-income individuals in Guangdong Province in 2021 (≤$1860, low income group; >$1860, middle-to-high income group) [27]. Then, we performed chi-square tests within the two sub-groups, to compare the vaccine uptake between pay-it-forward and standard-of-care arms. Multivariable logistic regressions were also conducted within the two sub-groups. It is theoretically possible that pay-it-forward might have a stronger association with vaccine uptake among individuals who were more financially incapable [28], [29].

**Fig. 1:**
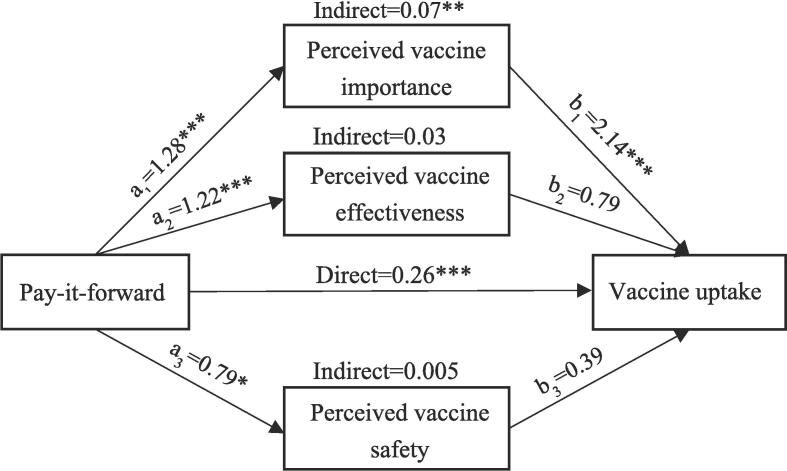
Mediation roles of perceived vaccine confidence (importance, effectiveness, safety) on the association between pay-it-forward and vaccine uptake.

Last, a mediation analysis was conducted to explore the indirect effect of vaccine confidence (vaccine importance, effectiveness and safety) on the association between pay-it-forward and vaccine uptake. In the parallel mediation model, we had the three vaccine confidence effects together as mediators in one model (Fig. 1). Pay-it-forward was the independent variable X, vaccine importance, effectiveness and safety were the mediator M_1_, M_2_ and M_3_, the vaccine uptake was the dependent variable Y. The paths a_1_ to a_3_ assess the relationship between X and M and paths b_1_ to b_3_ assess the relationship between M and Y. The direct effect of X on Y while partialling out the effect of M is denoted as path Direct. And the indirect effect of X on Y through M is the path Indirect. In our analysis results, the direct effect and indirect effects were reported after standardization [30]. The indirect effect is the product of standardized (a_1_ and b_1_)/ (a_2_ and b_2_)/ (a_3_ and b_3_).

Sociodemographic variables, including study site, age group, sex, age, educational level, occupation, annual income, marital status, cost-sensitivity, were controlled for the whole process of mediation analysis. Financial support, such as providing free vaccines, has a direct impact on vaccine uptake [31], so capability to pay (eg, proxy measure as annual income) were treated as a confounder but not a mediator. The “BruceR” package in R Studio was used to perform the mediation analysis and calculate the 95 % confidence interval using 10,000 bootstrapping resamples (R version 4.2.1).

## 3 Results

### 3.1 Descriptive outcomes

A total of 300 participants were enrolled in the study across two study arms (pay-it-forward, standard-of-care). The pay-it-forward arm had 150 participants, including 75 child caregivers and 75 older individuals with an average age of 53 years. The standard-of-care arm had 150 participants, with 75 child caregivers and 75 older individuals with an average age of 52 years. Of all participants, 73.3 % were female, and the vast majority were married or engaged (84 %). Over half of the participants (50.7 %) were unemployed, 76.3 % had received at least a middle school education, and 36.0 % earned under $1860 per year (low-income group). 66/300 (22 %) of the participants were sensitive to the cost (i.e., considered prices as a barrier). The samples' sociodemographic characteristics including age, sex, occupation, income, marital status and cost-sensitivity did not differ significantly between the two arms, except for their educational level (p = 0.04). We observed significant difference in participants’ vaccine confidence (influenza vaccine importance (p = 0.002), influenza vaccine safety (p < 0.001), and influenza vaccine effectiveness (p < 0.001) between participants in pay-it-forward arm and standard of care arm (Table 1).

**Table 1:** Sociodemographic characteristics and vaccine confidence level of participants in standard-of-care and pay-it-forward arms in Guangdong, 2020–21, n = 300.

	**Standard-of-care(N = 150)**	**Pay-it-forward(N = 150)**	**P value**
**Study site**			1.00
Yangshan	50(33.3)	50(33.3)	
Zengcheng	50(33.3)	50(33.3)	
Tianhe	50(33.3)	50(33.3)	
**Age group**			1.00
Child caregivers	75(50.0)	75(50.0)	
Older adults	75(50.0)	75(50.0)	
**Age (mean, SD)**	52.6(18.9)	51.6(17.1)	0.62
**Sex**			0.43
Male	37(24.7)	43(28.7)	
Female	113(75.3)	107(71.3)	
**Education completed**			0.04*
Primary school	41(27.3)	30(20.0)	
Middle school	76(50.7)	68(45.3)	
Undergraduate or college	33(22.0)	52(34.7)	
**Occupation**			0.70
Unemployed	73(48.7)	79(52.7)	
Peasant	20(13.3)	16(10.7)	
Employed	57(38.0)	55(36.7)	
**Individual annual income (US$)**			0.75
0 to 1860	57(38.0)	51(34.0)	
>1860 to 9300	51(34.0)	60(40.0)	
>9300 to 18,600	27(18.0)	26(17.3)	
>18600	15(10.0)	13(8.7)	
**Marital status**			1.00
Live alone	24(16.0)	24(16.0)	
Engaged or married	126(84.0)	126(84.0)	
**Cost-sensitivity**			0.58
Yes	35(23.3)	31(20.7)	
No	115(76.7)	119(79.3)	
**Influenza vaccine is important**			<0.001***
Agree	97(68.6)	127(88.2)	
Disagree	44(31.2)	17(11.8)	
**Influenza vaccine is safe**			0.002**
Agree	95(67.4)	120(83.3)	
Disagree	45(32.6)	24(16.7)	
**Influenza vaccine is effective**			<0.001***
Agree	88(62.4)	122(84.7)	
Disagree	53(37.6)	22(15.3)	

Note: * p < 0.05. ** p < 0.01. *** p < 0.001. Data are n (%) or mean (SD).

### 3.2 Multivariable analysis results

The pay-it-forward intervention was significantly associated with greater levels of perceived influenza vaccine importance (adjusted odds ratio (aOR) = 3.60, 95 %CI: 1.77–7.32), effectiveness (aOR = 3.37, 95 %CI: 1.75–6.52) and safety (aOR = 2.20, 95 %CI: 1.17–4.15). Greater perceived influenza vaccine importance was associated with increased vaccine uptake (aOR = 8.51, 95 %CI: 3.04–23.86) (Appendix Table 1).

### 3.3 Sub-group analysis

Table 2 showed that one-third of our participants belonged to the low-income sub-group according to local standards. The pay-it-forward intervention was associated with an increased odds of vaccine uptake (p < 0.001) compared against the self-paid strategy regardless of the income level of participants. Through sub-group analysis, people with greater perceived vaccine importance were more likely to receive the vaccine within both middle-to-high and low-income individuals (p = 0.002, aOR = 8.62, 95 %CI:2.23–33.39; p = 0.04, aOR = 6.08, 95 %CI:1.11–33.33) (Table 3). Additional sub-group analyses by level of cost-sensitivity were performed (Appendix Tables 2-3).

**Table 2:** Chi-square analysis to compare influenza vaccine uptake between standard-of-care and pay-it-forward arms in the two sub-groups, in Guangdong, 2020–2021.

Sub-groups		Vaccine uptake (n, %)	P
Individual annual income≤$1860 (N = 108)			<0.001***
	Standard-of-care (n = 57)	18(31.6)	
	Pay-it-forward (n = 51)	39(76.5)	
	Proportion difference	44.7	

Individual annual income>$1860 (N = 192)			<0.001***
	Standard-of-care (n = 93)	37(39.8)	
	Pay-it-forward (n = 99)	72(72.7)	
	Proportion difference	32.9	

Note: * *p* < 0.05. ** *p* < 0.01. *** *p* < 0.001.

**Table 3:** Multivariable logistic regression analysis to compare influenza vaccine uptake between standard-of-care and pay-it-forward arms in two sub-groups by income level, in Guangdong, 2020–2021.

	Vaccine uptake by annual individual income levels in USD			
	≤$1860 (N = 108)	P value	>$1860 (N = 192)	P value
**Intervention**				
Standard-of-care (reference)				
Pay-it-forward	4.90(1.27, 18.96)	0.02*	5.14(2.30, 11.48)	<0.001***

**Influenza vaccine is important**				
Disagree (reference)				
Agree	6.08(1.11, 33.33)	0.04*	8.62(2.23, 33.39)	0.002**

**Influenza vaccine is effective**				
Disagree (reference)				
Agree	2.83(0.66, 12.15)	0.16	2.14(0.71, 6.49)	0.18

**Influenza vaccine is safe**				
Disagree (reference)				
Agree	1.16(0.26, 5.31)	0.85	1.82(0.58, 5.69)	0.30

Note: The variables including age group, study site, sex, age, educational level, occupation, marital status, cost-sensitivity are not shown. * *p* < 0.05. ** *p* < 0.01. *** *p* < 0.001.

### 3.4 Mediation analysis results

Table 4 showed the coefficients of the parallel mediation model and single mediation models can be found in Appendix Table 4 and Appendix Figure panel 1. Both coefficients of a_1_ and b_1_ were significant at the 95 %confidence level. The indirect effect of the intervention of pay-it-forward on vaccination was significant mediated through confidence in vaccine importance (indirect effect_1_ = 0.07, 95 %CI: 0.02–0.11) and participants in the pay-it-forward arm were more likely to show higher confidence in vaccine importance which then subsequently leading to higher uptake compared to those in the standard-of-care. The coefficients of a_2_, a_3_ were significant at the 95 % confidence interval but b_2_ and b_3_ were not significant. The indirect effect of the intervention of pay-it-forward on vaccination, nevertheless, was non-significant through confidence in vaccine effectiveness and safety (indirect effect_2_ = 0.03, 95 %CI: −0.004–0.06; indirect effect_3_ = 0.005, 95 %CI: 0.02–0.03). Additionally, the direct effect of the pay-it-forward intervention on vaccination was statistically significant (direct effect = 0.26, 95 %CI: 0.15–0.37).

**Table 4:** Mediation roles of vaccine confidence on the association between pay-it-forward and vaccine uptake (parallel mediation model).

	Model4: Perceived vaccine importance & effectiveness & safety
Effect	Coefficient [95 % CI]
a_1_^i^	1.28***[0.59, 2.01]
a_2_	1.22***[0.57, 1.89]
a_3_	0.79*[0.16, 1.44]
b_1_	2.14***[1.16, 3.25]
b_2_	0.79[-0.07, 1.67]
b_3_	0.39[-0.50, 1.28]
Direct^ii^	0.26***[0.15, 0.37]
Indirect_1_iii	0.07**[0.02, 0.11]
Indirect_2_	0.03[-0.004, 0.06]
Indirect_3_	0.005[-0.02, 0.03]

Note: * *p* < 0.05. ** *p* < 0.01. *** *p* < 0.001.

Note: The direct effect was standardized. The indirect effect is the product of standardized a_1_ and b_1_/a_2_ and b_2_/ a_3_ and b_3_.

i : The coefficients a_1_ to a_3_ represent the association between pay-it-forward intervention and perceived vaccine importance, effectiveness and safety. And b_1_ to b_3_ represent the association between vaccine importance, effectiveness and safety and vaccine uptake. Coefficients are regression weights before standardization.

ii :The direct effect was completely standardized.

iii : The indirect effect is the product of standardized a_1_ and b_1_/a_2_ and b_2_/ a_3_ and b_3_.

## 4 Discussion

The parent intervention study suggested that the pay-it-forward strategy may increase influenza vaccine uptake compared to a self-paid standard approach [23]. However, in addition to the financial support that pay-it-forward offers, what other factors may be associated with increased influenza vaccine uptake is unclear. This study extends the pay-it-forward literature by testing our hypothesis that this intervention may work through changing service users’ vaccine confidence. This analysis identified the changing perceived vaccine importance as a significant mediator of the association between pay-it-forward intervention and improved vaccine uptake. This study also found that, regardless of individual income levels, pay-it-forward was associated with a remarkable increase in vaccine uptake compared to the standard-of-care approach.

We found that greater perceived influenza vaccine importance was associated with an increased uptake. This is consistent with previous study findings that participants' confidence in vaccine importance can positively impact vaccine uptake [25], [32], [33], [34]. A high level of public confidence in vaccine importance is key to achieving and maintaining a high coverage [33], [35]. Poor perceived vaccine importance is associated with greater vaccine skepticism and a decreased vaccination rate [25], [32], [34], whereas perceived vaccine importance may mitigate losses in vaccine uptake [25]. This is partly because perceived vaccine importance is a well-identified individual determinant of vaccine acceptance [34]. These indicate that health education and advocacy programs targeting improved public confidence in vaccine importance may be a first step in creating public demand and subsequently an increased uptake among priority groups.

Our data further suggested that pay-it-forward may be a solution that can help improve user-perceived vaccine importance, which subsequently led to an increased uptake. The parallel mediation analysis results showed that, amongst the three vaccine confidence domains, vaccine importance was identified as the only significant mediator of the association between the pay-it-forward intervention and vaccine uptake, and confidence in safety and effectiveness were found to be less relevant to the mediation pathway in our study sample. Our interpretation is that, in the Chinese market where there is still limited awareness and demand for influenza vaccines, pay-it-forward approach as an intervention package containing educational, peer-based psycho-behavioral, and community engagement components may help enhance public awareness and trust, perceived vaccine importance and subsequent acceptance of the vaccine [36], [37], [38]. This is supported by previous pay-it-forward studies suggesting that positive experiences (e.g., kindness and reciprocity generated among the community through donations and handwritten messages) contributed to community solidarity, public trust and encouraged people to receive medical service [39], [40], [41], [42], [43]. More in-depth data are however needed to better understand the mechanisms.

Finally, our sub-group regression analysis showed that, regardless of individual income level, pay-it-forward intervention may be associated with an increased odds of vaccine uptake compared against the self-paid strategy, and vaccine importance remained another significant factor associated with a higher level of vaccine uptake. These suggest that confidence in vaccine importance may be an important target domain for future interventions among varying income groups, and supported by our mediation model results, pay-it-forward may be a promising model to improve influenza vaccine uptake via improving perceived vaccine importance among different income groups. But future research is still needed to affirm potential causal effects.

## 5 Limitations

Our analysis has several limitations. First, the parent study used a quasi-experimental study design without randomization [44].Although the pay-it-forward participants were recruited immediately after standard-of-care group, reducing the influence of temporal changes on the observed differences, inferences made from this data should be made with caution [45]. More robust research, such as a randomized controlled trial, is needed to confirm this association. Our analysis informs a hypothesis to understand the potential mechanisms of a pay-it-forward intervention in vaccine services research. Second, education level appeared to differ between pay-it-forward and standard-of-care arms (Table 1), but this variable was adjusted in our regression and mediation models. Finally, participants from the sub-urban Zengcheng site had relatively higher levels of vaccine confidence compared to those from Yangshan and Tianhe (Appendix Table 1). We speculate that this might be because they had higher level of trust in the health facility but our current data lack relevant information to test the hypothesis and this needs further research to verify.

## 6 Implication

Our findings have important public health and research implications. Increasing perceived vaccine importance may help to increase vaccine uptake in the Chinese context. The pay-it-forward strategy increased vaccine uptake significantly and may have been mediated by confidence in vaccine importance. This study is pathbreaking in examining influenza-specific vaccine confidence among older adults and child caregivers in China, and leveraging an innovation to improve vaccine attitudes.

## 7 Conclusion

Our findings suggest that vaccine confidence is positively associated with pay-it-forward intervention. Perceived confidence in vaccine importance seems to be a potential mediator of the association between pay-it-forward and vaccine uptake. Regardless of the individual income level, pay-it-forward intervention may be a promising strategy to improve influenza vaccine uptake. The findings of our study suggest the possibility of a pro-social pay-it-forward model to increase influenza vaccine uptake among priority populations via improved perceived vaccine importance.

Author contributions

DW conceived the idea and analysis plan. WJ analyzed data and generated the figures and tables, XY offered help. WJ, CL and DW wrote the first draft of the paper and all coauthors provided constructive comments and edited the manuscript.

## Declaration of competing interest

The authors declare that they have no known competing financial interests or personal relationships that could have appeared to influence the work reported in this paper.

## Data Availability

The data will be available by putting on the website of WWW.SESHGlobal.ORG
